# The Springing of Plates in Soldering

**Published:** 1855-10

**Authors:** W. Calvert


### ARTICLE XII.

*The Springing of Plates in Soldering.* By W. CALVERT, D. D. S.

MUCH has already been said through the medium of our various dental journals and elewhere, upon this important and interesting subject—*i. e.* the springing of plates in soldering. And, notwithstanding all that has been said, the various opinions that have from time to time been advanced respecting this matter, nothing has as yet appeared, that was sufficiently authenticated to afford a safe and reliable *preventive* to this trying and vexatious matter.

The way in which this change is to be effectually obviated, *is* and ever has been to the dental profession a matter of deep interest, and demands of us investigation, in order that the application of proper means shall be made to secure right ends.

Now, the reason that plates spring in soldering, is unquestionably owing to the unequal expansibility of the materials constituting the case—comprising the metal, or plate and solder, the teeth, and the investment of plaster and sand. In all of these do we find different degrees of expansibility when exposed to the same heat, and unless they offer to each other, in a certain sense, an equal amount of resistance, there must result a change. As, for instance, in a case invested in plaster and sand, the combination of which would not expand and contract equally with the plate, upon being exposed to a heat requisite for soldering. The teeth, also, not expanding as much as the plate—being held together by the investment—are united by the stays to the plate, and in cooling, the unequal contraction of these different materials, prevent the plate from assuming its original form, and the result is a crooked or warped plate.

It has been, and I believe is yet contended, that in soldering, when the stays are made continuous, or even when the stays are isolated, but where the solder unite upon the surface of the plate, there *will be* a change in the plate—owing to the different contractibility of the plate and the solder. But this, I argue, is not necessarily so, for they act mechanically (that is by their resistance) towards each other. The one offering the greater resistance overbalancing the law governing the other. In the union of the stays to each other and to the plate, there can no change take place, unless there be an undue amount of solder thrown upon the plate—sufficient to overcome the resistance offered by it. On the contrary, (supposing that no *dentist*, now-a-days, would *heap* solder upon his operations,) the plate so resists the contraction of the solder, that it suspends, as it were, the particles of the metal, or, in other words, holds it extended; just as would be a piece of plate heated to redness, and while hot (and of course expanded) each end were suddenly and securely held. It now cools, and in consequence of the plate offering less resistance than is offered by the force applied at the ends of the plate, the cohesive attraction of the particles of the metal is overcome and the plate becomes permanently extended.

Now, a very easy and simple method (and one that I have satisfactorily tried) to ascertain whether a plate will spring except when an investment is thrown around it, is to strike up a plate in precisely the same manner as if teeth were to be mounted upon it, and after having it so prepared, after the swaging is completed, carefully anneal it; then prepare and fit a rim, or a number of pieces of stay plate, and solder upon the palatine surface of the plate, inside the alveolar ridge, occupying the same position as the stays of teeth should, and if ordinary care be taken, no change will take place. In order to satisfactorily prove this, it is only necessary after the swaging of the plate is completed, and before soldering on the rim or stays, to pour plaster into the plate, so as to obtain a correct cast, and after the soldering has been completed, replace the plate upon the cast, when it will at once be evident if any change has taken place.

Although there may be, and doubtless are, certain conditions which if not properly complied with, will increase the liability, if they do not absolutely produce the very thing we so much wish to avoid. Nevertheless, if what I have already attempted to set forth, be correct, the great *primary* cause of the springing of plates is owing to the combination of plaster and sand in the investment, or rather, I should say, to the excess of plaster.

Now, if a case of teeth were to be invested in something, equal in expansion and contraction with the plate, and be exposed to a requisite heat for soldering, it would be found that the teeth would be separated proportionably exactly with the expansion of the plate, the investment expanding equally. The teeth being here united by the stays to the plate, the case is left to cool; and in cooling the contraction being equal to the expansion, the teeth are brought back again by the investment and plate equally to just the position they left, and the result is that the plate resumes exactly its original form, or, *is not* sprung. But if the expansion of the plate should be greater than that of the investment, the teeth would (if not held close together) not be separated proportionately with the expansion of the plate, so that after the soldering, as the case cools, the the teeth would come together before the contraction of the plate was completed, and offering a greater amount of resistance than the plate was capable of equalizing or overcoming, would cause *it* to yield or spring.

I have already endeavored to indicate that the springing of plates is not due to the solder used in uniting the stays to each other and to the plate, and I have also attempted to show that it is almost wholly due to the investment, or at least, that the investment, or plaster and sand, is the *primary* cause of almost all the trouble.

I presume there are scarcely (I was about to say) two individuals that have adopted *any rule* by which to be governed in the use of these materials for investment, and the reason generally assigned is, that it is a matter of no importance, inasmuch as the excess of sand was merely a prevention to the cracking of the plaster. True, it is, that it does so act, and no less true is it, that it does more than this, as I trust I shall be presently able to show.

Let us now inquire into the relative expansion and contraction of the different materials which we have already been considering.

Both silver and gold, when heated to a degree sufficient to flow their respective solders, expand almost the same—the expansion of silver being about $\frac{17}{400}$ of an inch in three inches, and that of gold about $\frac{15}{400}$ of an inch in the same length.

Porcelain, or the material ordinarily used in the manufacture of teeth, expands but little over one-half as much as either silver or gold, the expansion being about $\frac{8}{400}$ of an inch in three inches.

Plaster alone, when exposed to heat, does not expand, or rather, I should say, by subjecting a piece of plaster of a given length, say three inches, to a heat sufficient for soldering, is found to contract one-twentieth of an inch, and in cooling contracts one-twentieth more, making a total contraction of one-tenth of an inch in three inches. When combined with sand in equal parts by measure, or by weight, one and a half ($1\frac{1}{2}$) ounces of plaster, and the three ounces of sand, and exposed to a heat equal with the above, contracts about $\frac{3}{100}$ of an inch in three inches, and expands only half that much.

When mixed in the proportion of four ounces of sand and one ounce of plaster it expands about $\frac{15}{400}$ of an inch, and contracts $\frac{16}{400}$ of an inch in three inches. When a still greater excess of sand is added, four ounces of sand to three-fourths of an ounce of plaster, the expansion and contraction are equal, expanding about $\frac{16}{400}$ of an inch in three inches. And when still more sand is added, the mean of several experiments, four ounces of sand and half an ounce of plaster is about $\frac{17\frac{1}{3}}{400}$ of an inch in three inches.

From the foregoing it would appear that the two last mentioned combinations of sand and plaster, compare favorably in expansion and contraction with silver and gold, and are therefore well suited for an investment—as also, a *prevention to the springing of plates.*

I have hinted that there were certain conditions to be met, aside from the investment always considered, in order to secure the most desirable results. I allude to the preparation of the plate, and properly heating up the case previous to the application of the flame of the blow-pipe to solder.

It is not only necessary that a plate should be frequently annealed during the preparation or swaging, but it is equally requisite that it should be thoroughly annealed *after the swaging* has been completed.

The plate having been thus thoroughly annealed, and the teeth properly adjusted to it, the case is ready to be placed in sand and plaster, and a very convenient way of doing this, is to have provided several open rings so as to be readily adapted to the shape and size of different cases. These may be made of tin or other plates, and about an inch in height, except that part of the ring intended to be posterior to the teeth and plate; it should be curved, not being more than about a quarter of an inch high in the middle. One of these rings sufficiently large being placed upon a flat surface, the sand and plaster may be mixed, and a portion of it placed in the ring and the plate bedded in it, allowing the cutting edges of the teeth to sink a little below the top of the ring. The space between the teeth and the ring may now be filled up as desired.

After the teeth have been backed, and the solder applied to the different parts to be united, the ring which has served to protect the sand and plaster may now be removed, and the case being dried is ready to solder.

For the purpose of heating up cases before soldering, it is best to be provided with a small sheet iron furnace, five or six inches high and about as much in diameter. Placing a live coal in the furnace, fill up even with the top or very nearly so with small pieces of charcoal. On the top of this lay the case to be heated, after which, place other pieces of charcoal around the outside of the case, in order that the heat shall be equally diffused over the whole case, then place it where there will be a good draught, and in a few minutes the entire case will be sufficiently hot to complete the soldering with the blow-pipe.

Since I have been in the habit of treating my operations as already described, I can in truth say, that I have not had the difficulties with which I previously had to contend, but, on the contrary, have had the most satisfactory results. And now, in conclusion, let me further add, that if the foregoing indications be strictly observed, there will be (shall I say it?) *no more springing of plates in soldering.—Dental News Letter.*

